# Efficacy and Safety of Tranexamic Acid in Emergency Trauma: A Systematic Review and Meta-Analysis

**DOI:** 10.3390/jcm10051030

**Published:** 2021-03-03

**Authors:** Mahdi Al-Jeabory, Lukasz Szarpak, Kecskes Attila, Michael Simpson, Adam Smereka, Aleksandra Gasecka, Wojciech Wieczorek, Michal Pruc, Maciej Koselak, Wladyslaw Gawel, Igor Checinski, Milosz J. Jaguszewski, Krzysztof J. Filipiak

**Affiliations:** 1 Outcomes Research Unit, Polish Society of Disaster Medicine, P.O. Box 78, 05-090 Raszyn, Poland; mmahdi@interia.pl (M.A.-J.); m.pruc@ptmk.org (M.P.); 2 Maria Sklodowska-Curie Bialystok Oncology Center, 15-027 Bialystok, Poland; 3 NATO Centre of Excellence for Military Medicine, 1555 Budapest, Hungary; train.nco@coemed.org; 4 Central Texas Regional SWAT, Leander, TX 78646, USA; mike@ps-med.com; 5 Department of Gastroenterology and Hepatology, Faculty of Medicine, Wroclaw Medical University, 50-367 Wroclaw, Poland; adam.smereka@umed.wroc.pl; 6 1st Chair and Department of Cardiology, Medical University of Warsaw, 02-091 Warsaw, Poland; aleksandra.gasecka@wum.edu.pl (A.G.); krzysztof.filipiak@wum.edu.pl (K.J.F.); 7 Department of Cardiology, University Medical Center Utrecht, 3584 CX Utrecht, The Netherlands; 8 Department of Emergency Medicine, Medical University of Warsaw, 02-091 Warsaw, Poland; w.wieczorek@easyrescue.pl; 9 Maria Sklodowska-Curie Medical Academy in Warsaw, 03-411 Warsaw, Poland; src.emergency@gmail.com; 10 Department of Surgery, The Silesian Hospital in Opava, 746 01 Opava, Czech Republic; w.b.gawel@gmail.com; 11 Department of Emergency Medical Service, Wroclaw Medical University, 50-367 Wroclaw, Poland; igor.checinski@umed.wroc.pl; 12 First Department of Cardiology, Medical University of Gdansk, 80-210 Gdansk, Poland; jamilosz@gmail.com

**Keywords:** tranexamic acid, trauma, bleeding, mortality, emergency medicine, systematic review, meta-analysis

## Abstract

In trauma patients, bleeding can lead to coagulopathy, hemorrhagic shock, and multiorgan failure, and therefore is of fundamental significance in regard to early morbidity. We conducted a meta-analysis to evaluate the efficacy and safety of tranexamic acid (TXA) in civil and military settings and its impact on in-hospital mortality (survival to hospital discharge or 30-day survival), intensive care unit and hospital length of stay, incidence of adverse events (myocardial infarct and neurological complications), and volume of blood product transfusion. The systematic review and meta-analysis were conducted according to the Preferred Reporting Items for Systematic Reviews and Meta-Analyses (PRISMA) guidelines. A systematic review of the literature using PubMed, Scopus, EMBASE, Web of Science, and the Cochrane Central Register and Controlled Trials (CENTRAL) database was conducted from inception to 10 January 2021. In-hospital mortality was reported in 14 studies and was 15.5% for the TXA group as compared with 16.4% for the non-TXA group (OR = 0.81, 95% CI 0.62–1.06, I^2^ = 83%, *p* = 0.12). In a civilian TXA application, in-hospital mortality in the TXA and non-TXA groups amounted to 15.0% and 17.1%, respectively (OR = 0.69, 95% CI 0.51–0.93, *p* = 0.02, I^2^ = 78%). A subgroup analysis of the randomized control trial (RCT) studies showed a statistically significant reduction in in-hospital mortality in the TXA group (14.3%) as compared with the non-TXA group (15.7%, OR = 0.89, 95% CI 0.83–0.96, *p* = 0.003, I^2^ = 0%). To summarize, TXA used in civilian application reduces in-hospital mortality. Application of TXA is beneficial for severely injured patients who undergoing shock and require massive blood transfusions. Patients who undergo treatment with TXA should be monitored for clinical signs of thromboembolism, since TXA is a standalone risk factor of a thromboembolic event and the D-dimers in traumatic patients are almost always elevated.

## 1. Introduction

Trauma is the leading cause of death in the population from 1 to 44 years old [1]. The main cause of early mortality in trauma patients is hemorrhage [2]. Bleeding initiates the cascade of reactions leading to coagulopathy and hemorrhagic shock [3,4], resulting in a higher occurrence of multiorgan failure as compared with patients without coagulopathy [5]. Although several mechanisms correlate with the risk of coagulopathy occurrence, for example, dilution and use of anticoagulative agents, due to the plethora of mechanism involved there are still no tangible factors that can be precisely responsible for the induction of coagulopathy [6]. While bleeding in a local wound presents little to no problem because it can usually be stopped by using compression [7], polytrauma patients with severe injury, for example, a broken pelvis, require far more attention and more sophisticated methods due to the lack of compression spots [8]. Therefore, we have focused on the use of tranexamic acid (TXA), a drug introduced as early as 1968 for menorrhagia treatment [9], which works by slowing down the conversion of plasminogen to plasmin, subsequently, reducing fibrinolysis and stabilizing the blood clot. The use of TXA, since 1968, has spread across the fields of medicine, including surgery [10], hematology [11] and most interestingly, trauma [12], due to its effectiveness in reducing both bleeding and mortality. The promising results and the lack of synthesis in the emergency setting of TXA administration have inspired us to conduct this meta-analysis on the safety and efficacy of TXA use in this environment. Therefore, we conducted a meta-analysis on the use of TXA in civil and military settings and its impact on in-hospital mortality (survival to hospital discharge or 30-day survival), ICU, hospital length of stay, and incidence of adverse events (myocardial infarct or central nervous system failure), as well as the effect of TXA on the volume of blood product transfusion.

## 2. Materials and Methods

This systematic review and meta-analysis were conducted according to the Preferred Reporting Items for Systematic Reviews and Meta-Analyses (PRISMA) guidelines [13]. For this meta-analysis, neither ethics committee approval nor patient consent were required.

### 2.1. Literature Search

A systematic review of the literature using PubMed, Scopus, EMBASE, Web of Science, and the Cochrane Central Register and Controlled Trials (CENTRAL) database was conducted from inception to 10 January 2021 with the following search strategy: “tranexamic acid” OR “tranexamic” OR “TXA” OR “hemorrhage control” AND “injuries*” OR “trauma” OR “wounds” AND “prehospital” OR “military” OR “combat” OR “civil*” OR “emergency medicine” OR “ER” OR “ED”. We also searched gray literature repositories such as Google Scholar. Finally, we manually retrieved and further reviewed references to TXA in eligible articles and systematic reviews.

### 2.2. Eligibility Criteria

Studies included in this meta-analysis fulfilled the following criteria (PICOS): (1) participants, patients with injury 18 years old or older; (2) intervention, tranexamic acid treatment; (3) comparison, non-TXA treatment; (4) outcomes, detailed information for survival; (5) study design, randomized controlled trials, quasi-randomized or observational studies comparing TXA and non-TXA care for their effects in patients with cardiac arrest. Studies were excluded if they were reviews, animal studies, case reports, letters, conference or poster abstracts, or articles not containing original data or focusing on brain injury.

### 2.3. Data Extraction

Raw data were extracted by using a standardized, premade form. We were careful to avoid inclusion of data from duplicate publications. In any case of suspected data discrepancies, we contacted the relevant author directly. Data extracted from eligible studies included the following characteristics: study and year, country, type of participants, number of participants, types of therapy, mortality rate, and adverse event occurrence. Two authors (M.A.-J. and W.W.) independently performed the literature search, study selection, and extraction of the baseline characteristics and outcome measures. Disagreements between the authors regarding values or analysis assignments were resolved through discussion with a third researcher (L.S.), and the decision was taken by the majority of the researchers.

### 2.4. Assessment of Risk of Bias

Two investigators (A.G. and L.S.) independently extracted individual study data and evaluated studies for risk of bias. Any disagreements were discussed and resolved in a consensus meeting with the third reviewer (M.J.J.). The ROBINS-I tool (tool to assess risk of bias in non-randomized studies of interventions) was used to assess the quality of non-randomized trials [14] and the RoB 2 tool (revised tool for risk of bias in randomized trials) was used to assess the quality of randomized studies [15]. The Robvis application was used to visualize risk of bias assessments [16]. The scale has seven main domains (confounding, participant selection, classification of interventions, deviation from interventions, missing data, outcome measurement, and selection of reported results) and assigns one point for each of the following four judgements: critical, serious, moderate, and low. The review authors’ judgments about each risk of bias item are provided in Figures S1–S4.

### 2.5. Outcomes and Subgroups

The primary outcome of the current meta-analysis was survival to hospital discharge or 30-day survival. The secondary outcomes were adverse events and other survival period rates. In addition, a subgroup analysis was performed with groups based on the civilian and combat applications of TXA.

### 2.6. Statistical Analysis

All statistical analyses were performed with Review Manager Software 5.4 (The Cochrane Collaboration, Oxford, Copenhagen, Denmark) [17]. The outcomes were summarized using the Mantel–Haenszel odds ratios (ODs) or mean differences (MDs). All results are presented with their 95% confidence interval (CI). When the continuous outcome was reported in a study as median, range, and interquartile range, we estimated means and standard deviations using the formula described by Hozo et al. [18]. Homogeneity of the effect size across trials was tested using the Cochrane Q statistic and the I2 statistic, which indicates the percentage of variability due to heterogeneity rather than sampling error [19]. A *p*-value <0.10 and I2 > 50% indicated heterogeneity, thus, helping to avoid false-negative results and the inclusion of such results in the meta-analysis. We performed sensitivity analysis using the Hartung–Knapp–Sidik–Jonkman method, when the number of studies was small (<10) [20]. Moreover, the random effects model was used for I2 > 50%; otherwise, the fixed effects model was employed. A *p*-value <0.05 was taken to indicate statistical significance [21]. Statistical testing was 2-tailed.

We looked for potential publication bias using a funnel plot if more than 10 trials were included for an outcome. For continuous outcomes, the Egger test was used to detect funnel plot asymmetry [22]. For dichotomous outcomes, we used the arcsine test. We considered publication bias to be present when the *p*-value was <0.1 in the asymmetry test. All analyses were performed using RevMan or Statistica 13.4EN (Tibco Inc., Tulsa, OK, USA).

## 3. Results

### 3.1. Characteristics of Studies Included in the Meta-Analysis

**Figure 1:**
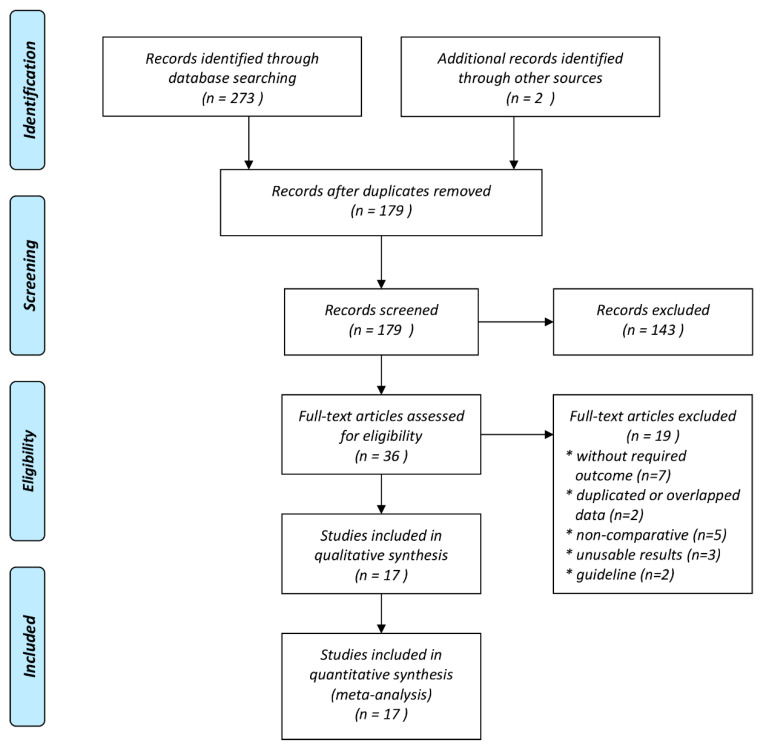
Meta-analysis flow chart of included and excluded studies.

The process of study selection is displayed in the flow chart of our study (Figure 1). In our initial electronic search, we identified 273 potential articles. Two studies were detected through manual scrutiny of reference lists of studies. After the removal of duplicates, we screened 118 articles by title and abstract for eligibility. From those studies, we only included thirty-seven trials for full-text evaluation. Finally, 17 studies were found to be eligible for quantitative analysis [23,24,25,26,27,28,29,30,31,32,33,34,35,36,37,38,39]. The details of the selected trials are summarized in Table 1 and Table S1. Among the seventeen studies, four studies were carried out in combat conditions [23,27,29,30], and 13 studies were carried out on civilian injuries [24,25,26,28,31,32,33,34,35,36,37,38,39]. Three studies were designed as randomized controlled trials [26,28,36]. The publication dates of these studies ranged from 2010 to 2020. The sample sizes of the included studies ranged from 40 to 20,207, with a total of 30,571 individuals, which altogether included patients with trauma treated with TXA (*n* = 14,413) or non-TXA (*n* = 16,158). Among the 17 articles, eight were conducted in USA [23,26,27,31,32,33,35,37,38], and one in each of the following countries: UK [24], Qatar [25], Iran [28], Israel [29], Afghanistan [30], Canada [34], and Germany [39]. One study was also conducted as a multi-country trial [36].

### 3.2. Primary Outcome

In-hospital mortality was reported in 14 studies and was 15.5% for the TXA group as compared with 16.4% for the non-TXA group (OR = 0.81, 95% CI 0.62–1.06, I2 = 83%, *p* = 0.12). The detailed characteristics of the causes of deaths are presented in Table S2.

**Figure 2:**
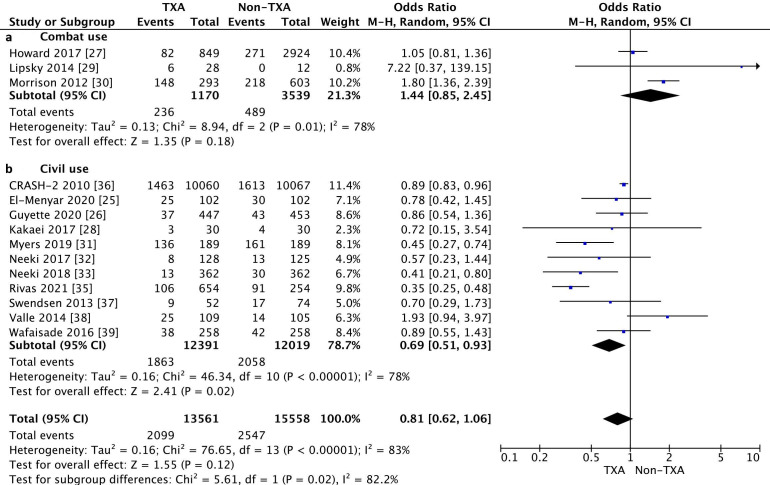
Forest plot of in-hospital mortality in the TXA group vs. non-TXA group. (**a**) Combat injuries; (**b**) Civilian injuries. The center of each square represents the weighted odds ratio (OR) for individual trials, and the corresponding horizontal line stands for the 95% confidence interval (CI). The diamonds represent pooled results.

The subgroup analysis showed that in-hospital mortality when TXA was used in combat conditions was 20.2% for the TXA group and 13.8% for the non-TXA group (OR 1.44, 95% CI 0.85–2.43, *p* = 0.18, I2 = 78%). In the case of civilian TXA application, in-hospital mortality in the TXA and non-TXA groups was statistically significantly differentiated and amounted to 15.0% and 17.1%, respectively (OR = 0.69, 95% CI 0.51–0.93, *p* = 0.02, I2 = 78%, Figure 2).

**Figure 3:**
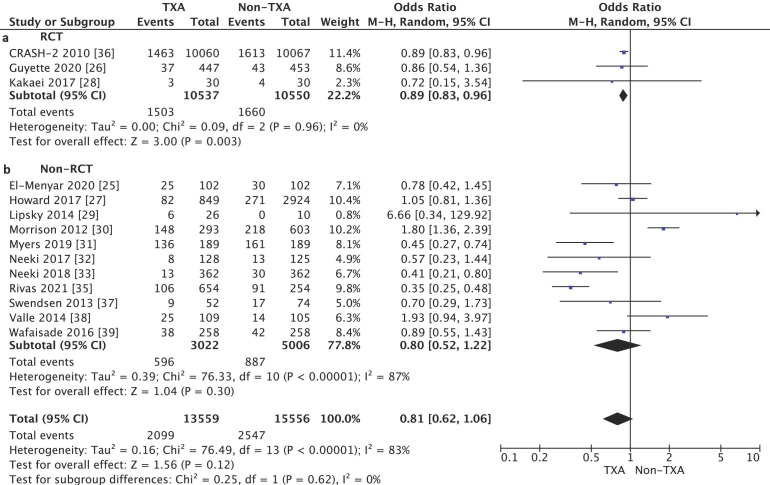
Forest plot of in-hospital mortality in the TXA group vs. the non-TXA group. (**a**) Randomized control trials (RCTs); (**b**) Non-randomized control trials. The center of each square represents the weighted odds ratio (OR) for individual trials, and the corresponding horizontal line stands for the 95% confidence interval (CI). The diamonds represent pooled results.

The subgroup analysis of the randomized control trial (RCT) studies showed a statistically significant reduction in in-hospital mortality in the TXA group (14.3%) as compared with the non-TXA group (15.7%) (OR = 0.89, 95% CI 0.83–0.96, *p* = 0.003, I2 = 0%, Figure 3). In the case of the non-RCT studies, no such relationship was found between the TXA and non-TXA groups (19.7% vs. 17.7%) (OR = 0.80, 95% CI 0.52–1.22, *p* = 0.30, I2 = 87%).

### 3.3. Secondary Outcomes

The risk of any vascular occlusive event in the TXA group was 1.8% as compared with 2.1% for the non-TXA group. The use of TXA as compared with non-TXA treatment was associated with a statistically significantly lower risk of an adverse event in the form of myocardial infarction (0.4% vs. 0.6%, respectively) or central nervous system failure (26.9% vs. 38.7% respectively), Table S3.

Additionally, the use of TXA was associated with a smaller volume of blood product transfusion as compared with the untreated patients (MD = −1.27, 95% CI −3.64–1.09, *p* = 0.29, I2 = 100%).

The length of hospital stay (LOS) in ICU was reported in seven studies (2693 patients). The mean LOS in ICU in the TXA group was 8.7 ± 11.2 days, and 7.0 ± 14.6 days for the non-TXA group (MD = 1.35, 95% CI −0.58–3.27, *p* = 0.17, I2 = 98%). Differences in ICU length of stay in the group of patients treated with TXA vs. non-TXA did not show statistical significance in both the case of combat injuries (MD = 0.12, 95% CI −5.0–5.31, *p* = 0.96, I2 = 11%) and in the case of civilian injuries (MD = 1.60, 95% CI −0.95–4.14, *p* = 0.22, I2 = 99%, Table S4).

Hospital LOS was reported by seven studies (2693 patients). The mean hospital LOS in the TXA group was 20.6 ± 24.5 days as compared with 17.2 ± 23.8 days for the non-TXA group (MD = 1.18, 95% CI −3.23–5.58, *p* = 0.60, I2 = 98%). Differences between hospital LOS in patients treated with TXA vs. non-TXA were not statistically significant in combat as well as in the case of civilian subgroups (MD = −18.80, 95% CI −46.04–8.44, *p* = 0.18) and in the case of civilian injuries (MD = 1.64, 95% CI −2.81–6.10, *p* = 0.47, I2 = 98%, Table S4).

### 3.4. Risk of Bias

A detailed description of the risk of bias assessment of the RCTs included in the meta-analysis is shown in Figures S4 and S5. The risk of bias assessment for the non-RCT studies is presented in Figures S6 and S7.

## 4. Discussion

Our meta-analysis revealed no statistically significant difference between the TXA and non-TXA administered groups when all patients were pooled. Patients treated within the civilian environment benefited from TXA administration and this group had a statistically significant lower mortality as compared with the patients treated without TXA. When we performed the subgroup analysis, we found that the mortality was lower only in the RCT environment, indicating that TXA should be given to the patients who exhibit the signs of severe shock and TXA administration should not be given based on physician discretion, while the TXA should be administered as soon as possible. Interestingly, in the military environment, the mortality, although not statistically significant, was higher in the TXA administrated group as compared with the non-TXA administered group. The explanation of this phenomenon is complex. In the combat environment, the ISS scores on admission for TXA administration were higher, indicating more severe trauma which correlated with the lower survival along with the higher risk of thrombus promotion, as presented by Ng et al. [40]. The study by Adair [23] further strengthened these findings as he showed that the soldiers who were given TXA had 3% increased odds of VTE and increased odds of PE, whereas the odds of DVT were found to be decreased.

Howard [27] also indicated that TXA administration in a combat environment can improve survival rate, however, it must be noted that the deployed medics were required to follow military Clinical Practice Guidelines [41] that indicate TXA must be given as part of a massive transfusion protocol, therefore, the sample of military patients who were administered with TXA was biased towards more severe injuries. A possible improvement in survival was proposed by Morrison [42] who analyzed the addition of cryoprecipitate to the TXA treatment and found that regardless of higher ISS, the group receiving the combined treatment had the lowest in-hospital mortality.

A major study by Cole [24] was the first to indicate the need to limit TXA treatment to patients who are in shock, since they are the only group that benefits from the TXA treatment.

The administration of TXA was found in our meta-analysis to be a protective factor against myocardial infarction in trauma patients. While the use of TXA in non-trauma patients was described in seven cases to correlate with myocardial infarction [43], the vast majority of “elective” TXA use describe it as a protective factor [44,45]. Additionally, the use of TXA was associated with a lower rate of central nervous failure [24], possibly via reducing the cytotoxicity in the TLR4/TNF axis [46].

Although not statistically significant, the use of TXA was associated with a lower need for blood product transfusion in trauma patients, especially the need for a massive transfusion. This finding applies for traumatic patients and also in the cases where a blood transfusion was part of the post-operative treatment, for example, hip surgeries or spine surgery [47].

The length of hospital stays, although not statistically significant, was longer in the TXA administered patients. This phenomenon can be explained by the higher ISS on admission, therefore, these patients required more medical interventions to save their lives. In contrast to the trauma patients, the administration of TXA in elective care reduces the length of stay [48,49].

In conclusion, it must be noted that TXA administration reduces mortality and morbidity in selected patients, while requiring intensive monitoring for the signs of thromboembolism.

### Limitations

A limitation of our study is that it excluded patients with head trauma, however, this allows for a better understanding of the pathogenesis and management of patients who have not suffered an injury to the head. Another limitation is the inclusion of retrospective analysis, which is lower in terms of data validity than a RCT; however, this allows for the broadening of the data pool, thus, resulting in higher data validity. The power of the study is also limited by the fact that the vast majority of the patients included in the analysis come from the CRASH-2 study.

## 5. Conclusions

The application of TXA is beneficial in severely injured patients, undergoing shock who require massive blood transfusions. Patients who undergo treatment with TXA should be monitored for clinical signs of thromboembolism, since TXA is a standalone risk factor of a thromboembolic event and the D-dimers in traumatic patients are almost always elevated.

## Figures and Tables

**Table 1:** Patient characteristics of the sixteen included studies.

Study	Country	Study Design	TXA Place of Infusion	TXA Group				Non-TXA Group			
				No.	Age	Sex, Male	ISS	No.	Age	Sex, Male	ISS
Adair et al., 2020 [23]	USA	Retrospective, observational analysis	Combat	318	24.5 ± 4.6	318 (100%)	NS	38	25.6 ± 5.2	38 (100%)	NS
Cole et al., 2020 [24]	UK	Prospective cohort study	Civil	160	42 ± 17.2	125 (78.1%)	33 ± 13	225	40 ± 18.6	185 (82.2%)	29 ± 10
Shakur et al., 2010 “CRASH-2” [36]	Multi-country	A randomized, placebo-controlled trial	Civil	10,093	34.6 ± 14.1	8439 (83.6%)	NS	10,114	34.5 ± 14.4	8496 (84.0%)	NS
El-Menyar et al., 2020 [25]	Qatar	Retrospective study	Civil	102	31.5 ± 0.8	98 (96.1%)	24.0 ± 0.8	102	31.5 ± 0.8	91 (89.2%)	24.99 ± 0.9
Guyette et al., 2020 “STAAMP” [26]	USA	Pragmatic, phase 3, multicenter double-blind placebo-controlled, superiority randomized clinical trial	Civil	447	41 ± 17	327 (73.2%)	13 (5–22)	456	42 ± 18	341 (74.8%)	11 (4–22)
Howard et al., 2017 [27]	USA	Retrospective study	Combat	849	24.8 ± 8.3	835 (98.4%)	21.5 ± 12.5	2924	25.1 ± 10.7	2794 (95.6%)	18.2 ± 11.2
Kakaei et al., 2017 [28]	Iran	Randomized-controlled trial	Civil	30	37.8 ± 10.1	23 (76.7%)	NS	30	35.8 ± 10.5	22 (73.3%)	NS
Lipsky et al., 2014 [29]	Israel	Retrospective study	Combat	28	28.25 ± 3.8	25 (89.3%)	17.8 ± 3.7	12	28.75 ± 5.5	10 (83.3%)	7 ± 2.3
Morrison et al., 2012 [30]	Afghanistan	Retrospective observational study	Combat	293	24.9 ± 9.6	285 (97.3%)	25.2 ± 16.6	603	23.1 ± 10.1	568 (94.2%)	22.5 ± 18.5
Myers et al., 2019 [31]	USA	Retrospective study	Civil	189	37.25 ± 4.8	142 (75.1%)	NS	189	35.25 ± 5.5	132 (69.8%)	NS
Neeki et al., 2017 [32]	USA	Multi-centered, prospective, observational cohort study with a retrospective chart-review comparison	Civil	128	38.23 ± 15.48	103 (80.5%)	12.96 ± 9.03	125	39.06 ± 16.66	104 (83.2%)	17 ± 10.74
Neeki et al., 2018 [33]	USA	Multi-centered, prospective, observational cohort study with a retrospective comparison	Civil	362	37.96 ± 16.11	293 (80.9%)	16.08 ± 10.69	362	37.64 ± 16.33	293 (80.9%)	17.15 ± 11.71
Ng et al., 2019 [34]	Canada	Retrospective study	Civil	67	42.3 ± 17.9	54 (80.6%)	27 ± 16	50	43.6 ± 20.4	42 (84.0%)	25 ± 16
Rivas et al., 2021 [35]	USA	A multicenter retrospective study	Civil	887	41 ± 18.1	480 (54.1%)	25 ± 3	446	40.3 ± 18.2	361 (80.9%)	27.3 ± 3.5
Swendsen et al., 2012 [37]	USA	Retrospective study	Civil	52	44.6 ± 20.3	37 (71.2%)	27.1 ± 15.0	74	47.6 ± 18.9	49 (66.2%)	20.5 ± 16.8
Valle et al., 2014 [38]	USA	Prospective study	Civil	150	43 ± 20	128 (85.3%)	28 ± 16	150	43 ± 20	129 (86.0%)	28 ± 17
Wafaisade et al., 2016 [39]	Germany	Retrospective study	Civil	258	43 ± 19	187 (72.5%)	24 ± 14	258	41 ± 18	187 (72.5%)	24 ± 16

NS, not specified; TXA, tranexamic acid; ISS, Injury severity score.

## Data Availability

Not applicable.

## Supplementary Materials

The following are available online at https://www.mdpi.com/2077-0383/10/5/1030/s1, Table S1: Characteristics of included studies, Table S2: In-hospital death by cause, Table S3: Adverse events, Table S4: Mechanism of injury, Table S5: Length of stay parameters, Figure S1. Forest plot of patients age in TXA vs. Control group; Figure S2. Forest plot of patient patients’ sex (male) in TXA vs. Control group; Figure S3. Forest plot of injury severity score at admission in TXA vs. Control group; Figure S4. A summary table of review authors’ judgements for each risk of bias item for each randomized study; Figure S5. A plot of the distribution of review authors’ judgements across randomized studies for each risk of bias item; Figure S6. A summary table of review authors’ judgements for each risk of bias item for each non-randomized study; Figure S7. A plot of the distribution of review authors’ judgements across non-randomized studies for each risk of bias item.
