# Supplementary material for: Efficacy and Safety of Tranexamic Acid in Emergency Trauma: A Systematic Review and Meta-Analysis
Source: J Clin Med. 2021 Mar 3;10(5):1030. doi: 10.3390/jcm10051030 (PMC7958951; doi:10.3390/jcm10051030)
Supplement: Supplementary file 1 [file jcm-10-01030-s001.pdf]

EFFICACY AND SAFETY OF TRANEXAMIC ACID IN TRAUMA PATIENTS IN EMERGENCY TRAUMA: A SYSTEMATIC REVIEW AND META-ANALYSIS

Supplementary File

CONTENT:

TABLE S1. CHARACTERISTICS OF INCLUDED STUDIES. 2

TABLE S2. IN-HOSPITAL DEATH BY CAUSE. 11

TABLE S3. ADVERSE EVENTS 11

TABLE S4. MECHANISM OF INJURY 12

TABLE S5. LENGTH OF STAY PARAMETERS 13

FIGURE S1. FOREST PLOT OF PATIENTS AGE IN TXA VS. CONTROL GROUP. 14

FIGURE S2. FOREST PLOT OF PATIENT PATIENTS' SEX (MALE) IN TXA VS. CONTROL GROUP 14

FIGURE S3. FOREST PLOT OF INJURY SEVERITY SCORE AT ADMISSION IN TXA VS. CONTROL GROUP. 15

FIGURE S4. A SUMMARY TABLE OF REVIEW AUTHORS' JUDGEMENTS FOR EACH RISK OF BIAS ITEM FOR EACH RANDOMIZED STUDY 15

FIGURE S5. A PLOT OF THE DISTRIBUTION OF REVIEW AUTHORS' JUDGEMENTS ACROSS RANDOMIZED STUDIES FOR EACH RISK OF BIAS ITEM. 16

FIGURE S6. A SUMMARY TABLE OF REVIEW AUTHORS' JUDGEMENTS FOR EACH RISK OF BIAS ITEM FOR EACH NON-RANDOMIZED STUDY. 16

FIGURE S7. A PLOT OF THE DISTRIBUTION OF REVIEW AUTHORS' JUDGEMENTS ACROSS NON-RANDOMIZED STUDIES FOR EACH RISK OF BIAS ITEM. 17

Table S1. Characteristics of included studies.

Study	Inclusion criteria	Exclusion criteria	TXA treatment	Primary outcome(s)	Findings
Adair et al. 2020	Trauma patients had both an injury severity score (ISS) ≥10 and a massive transfusion (MT).	Patients who did not meet the criteria for a MT.	NS	The occurrence of a VTE during the patient's course of treatment.	TXA use with an ISS >10 and MT resuscitation had 3% increased odds of venous thromboembolism (VTE) and an increased odd of pulmonary embolism, whereas the odds of deep vein thrombosis were found to be decreased after multiple imputation analysis. Further research on the long-term risks and benefits of TXA usage in the military population is recommended.
Boutonnet et al. 2020	Adult (>16 years of age) trauma patients admitted directly to one of the trauma centers. Patients were included if they had (1) presented a major hemorrhage (defined by the need of four or more packed red blood cells [pRBC] in the first 6 hours following the trauma), or (2) received at least one pRBC in the emergency room (ER), or (3) received vasopressors either in the prehospital setting or in the ER.	Not meet inclusion criteria.	NS	Hospital mortality.	The use of TXA in the management of severely injured trauma patients, in a mature trauma care system, was not associated with reduction in the hospital mortality. An independent association with a better survival was found in a selected population of patients requiring pRBC transfusion in the ER.
Cole et al. 2020	All adult trauma patients (>15 years) admitted to the critical care unit following trauma team activation.	Retrospectively exclusion patients found to have an injury severity score (ISS) less than 15.	1 g in the first 3 hours after injury followed by a 1 g infusion over 8 hours.	48 hours or less (early) and more than 48 hours (late) mortality, organ failure, presence of infection, episodes	TXA as part of a major hemorrhage protocol within a mature civilian trauma system provides outcome benefits specifically for severely injured shocked patients.

				of venous thromboembolism (VTE), episodes of stroke and myocardial infarction, ventilator-free days (VFDs), critical care length of stay (LOS), and total hospital LOS	
Shakur et al. 2010 “CRASH-2”	All trauma patients with ongoing significant hemorrhage (systolic blood pressure less than 90 mmHg and/or heart rate more than 110 beats per minute), or who are considered to be at risk of significant hemorrhage, and are within 8 hours of the injury, are eligible for trial entry if they appear to be at least 16 years old. Although entry is allowed up to 8 hours from injury, the earlier that patients can be treated the better.	The fundamental eligibility criterion is the responsible doctor's 'uncertainty' as to whether or not to use an antifibrinolytic agent in a particular adult with traumatic hemorrhage. Patients for whom the responsible doctor considers there is a clear indication for antifibrinolytic therapy should not be randomized. Likewise, patients for whom there is considered to be a clear contraindication to antifibrinolytic therapy (such as, perhaps, those who have clinical evidence of a thrombotic disseminated intravascular coagulation) should not be randomized. Where the responsible doctor is substantially uncertain as to whether or not to use an antifibrinolytic, all these patients are eligible for randomization and should be	1 g of tranexamic acid infused over 10 min, followed by an intravenous infusion of 1 g over 8 h, or matching placebo (0·9% saline).	Death in hospital within 4 weeks of injury.	Tranexamic acid safely reduced the risk of death in bleeding trauma patients in this study. On the basis of these results, tranexamic acid should be considered for use in bleeding trauma patients.

		considered for the trial. There are no other pre-specified exclusion criteria			
El-Menyar et al. 2020	All patients of both genders aged 16 to 80 years old treated with TXA during the prehospital phase.	All patients receiving the first dose of TXA at the ED. Patients <16 and >80 years of age, vulnerable population (prisoners and pregnant patients), traumatic brain injury with exposed brain, isolated drowning or hanging victims.	1-g in the first 3 hours after injury followed by a 1 g infusion over 8 hours.	Patient characteristics.	Prehospital TXA administration is associated with less in-hospital blood transfusion and massive transfusion protocol (MTP). There is no significant increase in the thromboembolic events and mortality.
Ghawnni et al. 2018	Patients 16 years of age or older who met one or more of the following criteria: (1) tachycardia (defined as a heart rate [HR] ≥110 beats per minute on arrival to the emergency department [ED]); (2) hypo- tension (defined as a systolic blood pressure [SBP] ≤90 on ED arrival); and/or (3) requiring at least 1 unit of PRBCs in the ED.	Patients who received TXA at a peripheral hospital.	1g over 10 min followed by an infusion of 1 g over 8 hours.	The compliance rate of TXA administration.	Compliance with TXA administration to trauma patients with suspected major bleeding was low. Quality improvement strategies aimed at increasing appropriate use of TXA are warranted.
Guyette et al. 2020 "STAAMP"	Injured patients at risk for hemorrhage transported from the scene or transferred from an outside emergency department to a participating site within an estimated 2 hours of the time of injury were eligible for enrollment if they experienced at least 1 episode of hypotension (systolic blood pressure ≤90 mm Hg) or tachycardia (heart rate ≥110 beats per minute) before arrival at a participating center.	Age older than 90 years or younger than 18 years, lack of intravenous or intraosseous access, isolated fall from standing, documented cervical cord injury, known prisoner or pregnancy, traumatic arrest of more than 5 minutes, penetrating brain injury, isolated drowning or hanging, objection to study voiced at scene, or wearing a STAAMP study opt-out bracelet.	1-g of tranexamic acid infused during 8 hours, or a bolus of 1-g of tranexamic acid followed by 1-g of tranexamic acid infused during 8 hours	30-day mortality.	In injured patients at risk for hemorrhage, tranexamic acid administered before hospitalization did not result in significantly lower 30-day mortality. The prehospital administration of tranexamic acid after injury did not result in a higher incidence of thrombotic complications or adverse events. Tranexamic acid given to injured patients at risk for hemorrhage in the prehospital setting is safe and

					associated with survival benefit in specific subgroups of patients.
Howard et al. 2017	Patients had to have been injured in combat in Afghanistan, been admitted to a role 3 MTF, and received a blood transfusion of at least one unit.	NS	NS	All-cause mortality and the occurrence of PE or DVT.	In the largest study on TXA use in a combat trauma population, TXAwas not significantly associated with mortality, due to lack of statistical power. However, our HR estimates for mortality among patients who received TXA are consistent with previous findings from the CRASH-2 trial. At the same time, continued scrutiny and surveillance of TXA use in military trauma, specifically for prevention of thromboembolic events, is warranted.
Kakaei et al. 2017	Patients between 15-50 years of age, systolic blood pressure (SBP) less than 90 mmHg or heart rate more than 110 per min or both, trauma to admission interval less than eight hours, and not being in need of emergent surgical intervention.	Patients younger than 15 years of age, patients older than 50 years of age, having contraindication to receive Tranexamic acid (pregnancy, known thromboembolic events, defective color vision, history of vascular occlusive disease, hyper-coagulopathy, history of allergic reaction and history of angioedema), and being in need of emergent surgical intervention.	1-g dose (two 500 mg vials) infused by 100 ml of saline. Then, another 1 g dose was administered during eight hours.	The outcomes of patients were assessed during hospital admission, discharge, and one-month after their admission. Duration of admission, intensive care unit (ICU) stay and mortalities were recorded and assessed in study patients.	Tranexamic acid is safe and effective in reducing platelet transfusion rate in patients with trauma-related significant hemorrhage. However, transfusion need and mortality would not reduce by its use in trauma patients.
Lipsky et al. 2014	Any penetrating injury to the torso, including the neck, axillae, groin, and buttocks. Blunt or penetrating injury accompanied by signs of shock. Shock was	NS	Adult casualties are given 1-g IV, either by slow push (5–10 min) or mixed with crystalloid for	Patient characteristics.	TXA should be considered a viable option for use by advanced life support providers at the point of injury, provided that evacuation is not delayed, as part of ongoing

	defined as the presence of any of the following: systolic blood pressure (SBP) <90 mmHg, heart rate (HR) >100 beats per minute on repeated measurement, delayed capillary refill (>2 s), or altered level of consciousness in a casualty without blunt head trauma. This definition of shock was chosen to be consistent with the IDF fluid resuscitation protocol for trauma [8]. If shock is diagnosed, TXA is given even if haemorrhage has ceased.		infusion. If the casualty is in the field for an extended period (i.e., greater than 3 h) due to delayed evacuation, a second 1-g dose of TXA is given 3 h after the initial dose. In exceptional circumstances (i.e., where obtaining IV access would delay transport or in a mass casualty setting), TXA may be given orally at a dose of 1.5-g. TXA is given at the end of the secondary survey with other treatment adjuncts such as analgesics and antibiotics.		efforts to improve survival among hemorrhaging patients.
Morrison et al. 2012	Patients who received at least 1 unit of PRBCs within 24 hours of admission following combat-related injury	NS	Intravenous bolus of 1 g, repeated as felt indicated by the managing clinician.	24 and 48 hours and in-hospital mortality.	The use of TXA with blood component based resuscitation following combat injury results in improved measures of coagulopathy and survival, a benefit that is most prominent in patients requiring massive transfusion. Treatment with TXA should be implemented into clinical practice as part of a resuscitation strategy following severe wartime injury and hemorrhage.

Myers et al. 2019	Trauma patients undergoing air medical transport to one of the participating centers within 2 hours of injury, with systolic blood pressure (SBP) < 90 mmHg and heart rate (HR) >110 bpm (SBP and HR criteria need not be simultaneous)	Patients taking prehospital anticoagulation, those with a known history of DVT or PE, or hereditary coagulopathy, and those who had received prehospital TXA as part of a separate trial protocol.	1-g of TXA.	The occurrence of a VTE during the patient's course of treatment.	Tranexamic acid may be an independent risk factor for VTE. Future investigation is needed to identify which patients benefit most from TXA, especially given the risks of this intervention to allow a more individualized treatment approach that maximizes benefits and mitigates potential harms.
Neeki et al. 2017	The prehospital and hospital use of TXA should be considered for all trauma patients that meet any of the following criteria:<br>•Blunt or penetrating trauma with signs and symptoms of hemorrhagic shock<br>•Systolic blood pressure of less than 90 mmHg at scene of injury, during air and/or ground medical transport, or upon arrival to designated trauma centers<br>•Any sustained blunt or penetrating injury within three hours<br>•Patients who are considered to be high risk for significant hemorrhage<br>• Estimated blood loss of 500 milliliters in the field accompanied with a heart rate >120<br>o Bleeding not controlled by direct pressure or tourniquet	•Any patient <18 years of age<br>•Any patient with an active thromboembolic event (within the last 24 hours) – i.e. active stroke, myocardial infarction or pulmonary embolism<br>•Any patient with a hypersensitivity or anaphylactic reaction to TXA<br>•Any patient more than three hours post-injury<br>•Traumatic arrest with more than five minutes of cardiopulmonary resuscitation without return of vital signs<br>•Penetrating cranial injury<br>•Traumatic brain injury with brain matter exposed<br>•Isolated drowning or hanging victims<br>•Documented cervical cord injury with motor deficits	One gram of TXA in 100 ml of 0.9% normal saline infused over 10 minutes via intravenous or intraosseous access.	Mortality, measured at 24 hours, 48 hours, and 28 days.	Preliminary evidence from the Cal-PAT study suggests that TXA administration may be safe in the prehospital setting with no significant change in adverse events observed and an associated decreased use of blood products in cases of trauma-induced hemorrhagic shock. Given the current sample size, a statistically significant decrease in mortality was not observed. Additionally, this study demonstrates that it may be feasible for paramedics to identify and safely administer TXA in the prehospital setting.

	Major amputation of any extremity above the wrists and above the ankles				
Neeki et al. 2018	The prehospital and hospital use of TXA should be considered for all trauma patients that meet any of the following criteria:<br>•Blunt or penetrating trauma with signs and symptoms of hemorrhagic shock within three hours of injury.<br>-Systolic blood pressure of less than 90 mmHg at scene of injury, during air and/or ground medical transport, or upon arrival to designated trauma centers.<br>-Heart rate > 120.<br>-Estimated blood loss of 500 milliliters in the field.<br>-Bleeding not controlled by direct pressure or tourniquet.<br>•Major amputation of any extremity above the wrists and above the ankles.	•Any patient <18 years of age.<br>•Any patient more than three hours post-injury.<br>•Any patient with an active thromboembolic event (within the last 24 hours) – i.e., active stroke, myocardial infarction or pulmonary embolism.<br>•Any patient with a hypersensitivity or anaphylactic reaction to TXA.<br>•Traumatic arrest with more than five minutes of cardiopulmonary resuscitation without return of vital signs.<br>•Penetrating cranial injury.<br>•Traumatic brain injury with brain matter exposed.<br>•Isolated drowning or hanging victims.<br>•Documented cervical cord injury with motor deficits.	1-gof TXA in 100 ml of 0.9% normal saline infused over 10 minutes via intravenous (IV) or intraosseous access.	Mortality measured at 24 hours, 48 hours, and 28 days.	Findings from the Cal-PAT study suggest that TXA use in the civilian prehospital setting may safely improve survival outcomes in patients who have sustained traumatic injury with signs of hemorrhagic shock.
Ng et al. 2019	Trauma patient >16 years of age, significant hemorrhage defined as systolic blood pressure <90 mm Hg and/or heart rate >110 bpm, and presentation within 8 h of injury.	Patients with a history of cardiovascular disease, thromboembolic events, bleeding diathesis, renal failure with Cr > 250micromol/L, or those that were pregnant or on anticoagulants.	1-g IV/10 min bolus and 1-g IV/8 h infusion.	Proportion of trauma patients that met indication criteria for TXA; proportion that received both doses as per CRASH-2 protocol (1 g IV/10 min bolus and 1 g IV/8	<10% of adult trauma patients that met the indication for TXA received it according to the CRASH-2 protocol. Of those patients that received TXA, 76% did so within 3 h.

				h infusion); proportion that received a pre-hospital dose; and for those that received TXA, proportion that received the medication within 1 h, 1 to 3 h, or N3 h from time of injury	
Rivas et al. 2021	All injured patients ages 18–80 years old, presenting directly to the trauma center from the scene, and receiving at least 5 units of packed red blood cells (PRBC) within the first 24 h of hospital arrival.	Patients transferred from a referring hospital, died within 24 h of hospital admission, were pregnant, received TXA more than 3 h following injury or lacked documentation regarding timing of TXA administration.	NS	The incidence of VTE, defined as a composite measure of duplex confirmed DVT and computerized tomography angiogram confirmed pulmonary embolism, as well as the individual incidence of DVT or pulmonary embolism during the entire hospital stay.	
Swendsen et al. 2012	Trauma patients 18 years or older who met triage criteria for serious injury and at least one of the following: 1) hypotension (systolic blood pressure <90 mm Hg) upon presentation, 2) massive transfusion guideline	Patients who were transferred from another hospital or injured more than 3 hours previously.	1-gr bolus infusion over 10 minutes followed by a 1-g infusion over 8 hours.	Death within 24 hours; death during hospitalization; venous thromboembolic events (VTE) (deep	In civilian trauma, early TXA administration confers early survival advantage without affecting blood product usage but may increase the risk of DVT/PE and AKI.

	activation in the Emergency Department (ED), or 3) transport directly to the operating room (OR) or interventional radiology (IR) suite from the ED.			vein thrombosis or pulmonary embolism); myocardial infarction (MI); stroke; acute kidney injury (AKI); and blood product usage.	
Valle et al. 2014	All adult patients who underwent emergency OR directly from the resuscitation area were prospectively entered into a registry.	OR for isolated orthopedic and/or neurosurgical indications and minor trauma operations such as those for complex wound closures.	1-g bolus intravenously administered, followed by a 1-g infusion over 8 hours, starting within 3 hours of admission.	Patient characteristics.	For the highest injury acuity patients, TXA was associated with increased, rather than reduced, mortality, no matter what time it was administered. This lack of benefit can probably be attributed to the rapid availability of fluids and emergency OR at this trauma center. Prospective studies are needed to further identify conditions that may override the benefits from TXA.
Wafaisade et al. 2016	ADAC Air Rescue Service database: a) Primarily admitted trauma patient; b) Critical injury, defined as preclinically assessed NACA IV (potentially life-threatening), NACA V (acute danger) or NACA VI (respiratory and/or cardiac arrest); c) Admission to a trauma center participating in the TR-DGU<br>2. TR-DGU database: a) Primary admission; b) Treatment in a German trauma center (i.e.,	NS	1 g of tranexamic acid infused over 10 min, followed by an intravenous infusion of 1 g over 8 h, or matching placebo (0·9% saline).	Laboratory findings including data on transfusions, and outcomes.	This is the first civilian study, to our knowledge, in which the effect of prehospital TXA use in trauma patients has been examined. TXA was associated with prolonged time to death and significantly improved early survival.

	exclusion of trauma centers from other countries)				

Legend: MT = Massive transfusion defined as having received ≥10 units of packed red blood cells (PRBC) and/or whole blood (WB) in the first 24 hours after injury; TXA = tranexamic acid; VTE = venous thromboembolism; NS = Not specified.

Table S2. In-hospital death by cause.

Cause of death	No. of studies	Events in TXA group	Events in Control group	OR (95%CI)	P value	I^2 statistic
Bleeding	2	491/10,112 (4.9%)	582/10,141 (5.7%)	0.75 (0.40, 1.39)	0.36	25%
Vascular occlusion (includes MI, stroke, and pulmonary embolism)	2	33/10,112 (0.3%)	50/10,141 (0.5%)	0.67 (0.43, 1.05)	0.08	0%
Multiorgan failure	1	209/10,060 (2.1%)	233/10,067 (2.3%)	0.90 (0.74, 1.08)	0.25	NA
Head injury	2	609/10,112 (6.0%)	624/10,141 (6.2%)	0.98 (0.87, 1.10)	0.70	60%
Other causes	1	129/10,060 (1.3%)	137/10,067 (1.4%)	0.94 (0.74, 1.20)	0.63	NA

Legend: CI = Confidence interval; MI = Myocardial infarction; OR = Odds ratio; TXA = Tranexamic acid;

Table S3. Adverse events

Cause of death	No. of studies	Events in TXA group	Events in Control group	OR (95%CI)	P value	I^2 statistic
Myocardial infarction	5	45/11,288 (0.4%)	64/10,982 (0.6%)	0.66 (0.45, 0.97)	0.03	0%
Stroke	5	73/11,288 0.6%)	76/10,982 (0.7%)	0.90 (0.65, 1.24)	0.50	40%

Thromboembolic events	6	67/1,308 (5.1%)	62/963 (6.4%)	0.89 (0.37, 2.11)	0.79	60%
Pulmonary embolism	5	137/12,112 (1.1%)	117/13,800 (0.8%)	1.57 (0.79, 3.13)	0.20	80%
Deep vein thrombosis	6	105/12,240 (0.9%)	105/13,925 (0.8%)	1.13 (0.51, 2.51)	0.77	83%
Coagulation failure	1	5/160 (3.1%)	5/225 (2.2%)	1.42 (0.40, 4.99)	0.58	NA
Multiple organ failure	3	106/681 (15.6%)	156/799 (19.5%)	0.87 (0.66, 1.16)	0.35	39%
Acute kidney failure	2	22/212 (10.4%)	17/799 (2.1%)	1.97 (1.01, 3.86)	0.05	0%
Hepatic failure	1	5/160 (3.1%)	2/225 (0.9%)	1.21 (0.81, 1.82)	0.35	NA
Sepsis	1	4/67 (6.0%)	8/119 (6.7%)	0.88 (0.26, 3.04)	0.84	NA
Infection	1	89/160 (55.6%)	113/225 (50.2%)	1.24 (0.83, 1.87)	0.30	NA

Legend: CI = Confidence interval; OR = Odds ratio; TXA = Tranexamic acid;

Table S4. Mechanism of injury

Mechanism of injury	No. of studies	Events in TXA group	Events in Control group	OR (95%CI)	P value	I^2 statistic
Penetrating	5	637/1,782 (35.7%)	306/1,054 (29.0%)	1.23 (0.61, 2.47)	0.57	87%
Blunt	2	670/1205 (55.6%)	257/484 (53.1%)	1.35 (0.42, 4.33)	0.61	71%
Burn	1	5/318 (1.6%)	2/38 (5.3%)	0.29 (0.05, 1.54)	0.14	NA

Legend: CI = Confidence interval; OR = Odds ratio; TXA = Tranexamic acid;

Table S5. Length of stay parameters

Cause of death	No. of studies	Mean time in TXA group	Mean time in Control group	MD (95%CI)	P value	I^2 statistic
**ICU length of stay**						
Combat use	2	12.2 ± 13.3	19.7 ± 61.4	0.12 (-4.07, 5.31)	0.96	11%
Civil use	6	10.9 ± 10.0	7.0 ± 7.2	2.62 (-0.58, 5.86)	0.11	100%
**Hospital length of stay**						
Combat use	1	34.7 ± 35.1	53.5 ± 84.8	-18.80 (-46.04, 8.44)	0.18	NA
Civil use	7	15.9 ± 16.9	16.2 ± 16.2	0.65 (-2.25, 3.54)	0.66	98%

Legend: CI = Confidence interval; MD = Mean difference; TXA = Tranexamic acid;

Study or Subgroup	TXA Mean	TXA SD	TXA Total	Control Mean	Control SD	Control Total	Weight	Mean Difference IV, Fixed, 95% CI
Adair 2020	24.5	4.6	318	25.6	5.2	38	1.1%	−1.10 [−2.83, 0.63]
Cole 2015	42	17.2	160	40	18.6	225	0.3%	2.00 [−1.61, 5.61]
CRASH−2 2010	34.6	14.1	10093	34.5	14.4	10114	21.9%	0.10 [−0.29, 0.49]
El−Menyar 2020	31.5	0.8	102	31.5	0.8	102	70.3%	0.00 [−0.22, 0.22]
Guyette 2020	41	17	447	42	18	456	0.6%	−1.00 [−3.28, 1.28]
Kakaei 2017	37.8	10.1	30	35.8	10.5	30	0.1%	2.00 [−3.21, 7.21]
Lipsky 2014	28.25	3.8	27	28.75	5.5	10	0.2%	−0.50 [−4.20, 3.20]
Myers 2019	37.25	4.8	189	35.25	5.5	189	3.1%	2.00 [0.96, 3.04]
Neeki 2017	38.23	15.48	128	39.06	16.66	125	0.2%	−0.83 [−4.80, 3.14]
Neeki 2018	37.96	16.11	362	37.64	16.33	362	0.6%	0.32 [−2.04, 2.68]
Ng 2019	42.3	17.9	67	43.6	20.4	50	0.1%	−1.30 [−8.40, 5.80]
Rivas 2021	41	18.1	887	40.3	18.2	446	0.8%	0.70 [−1.37, 2.77]
Swendsen 2013	44.6	20.3	52	47.6	18.9	74	0.1%	−3.00 [−10.00, 4.00]
Valle 2014	42	20	150	43	20	150	0.2%	−1.00 [−5.53, 3.53]
Wafaisade 2016	43	19	258	41	18	258	0.3%	2.00 [−1.19, 5.19]
**Total (95% CI)**			**13270**			**12629**	**100.0%**	**0.08 [−0.10, 0.26]**

Heterogeneity: $Chi^2 = 21.03$, df = 14 (P = 0.10); $I^2 = 33\%$
Test for overall effect: Z = 0.85 (P = 0.40)

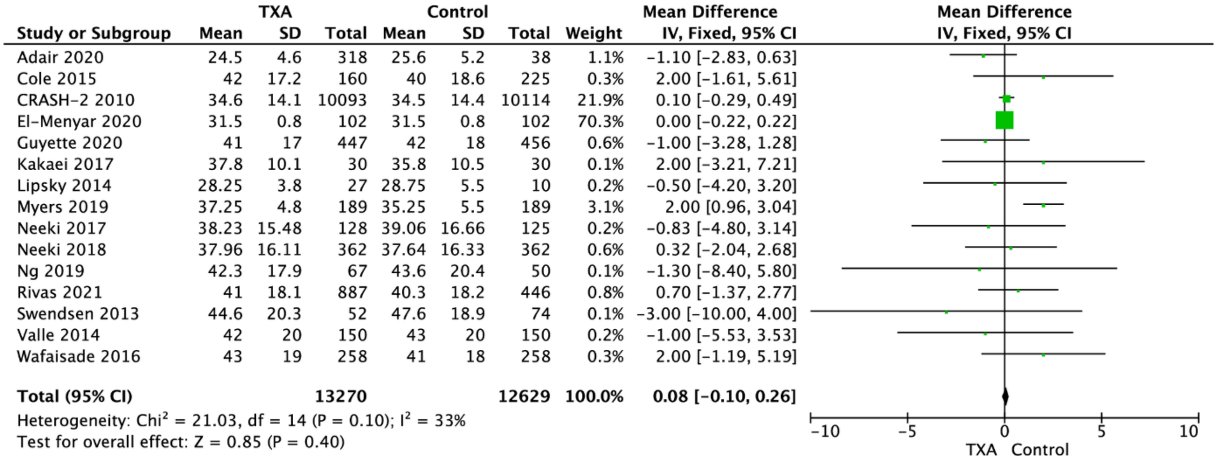

Figure S1. Forest plot of patients age in TXA vs. Control group. The center of each square represents the weighted mean differences for individual trials, and the corresponding horizontal line stands for a 95% confidence interval. The diamonds represent pooled results.

Study or Subgroup	TXA Events	TXA Total	Control Events	Control Total	Weight	Odds Ratio M−H, Fixed, 95% CI
Adair 2020	318	318	38	38		Not estimable
Cole 2015	78	160	82	225	1.8%	1.66 [1.10, 2.50]
CRASH−2 2010	8439	10093	8496	10114	71.0%	0.97 [0.90, 1.05]
El−Menyar 2020	98	102	91	102	0.2%	2.96 [0.91, 9.63]
Guyette 2020	327	447	341	456	4.6%	0.92 [0.68, 1.24]
Kakaei 2017	23	30	22	30	0.3%	1.19 [0.37, 3.85]
Lipsky 2014	25	28	10	12	0.1%	1.67 [0.24, 11.52]
Morrison 2012	285	293	568	603	0.5%	2.20 [1.01, 4.79]
Myers 2019	142	189	132	189	1.7%	1.30 [0.83, 2.05]
Neeki 2017	103	128	104	125	1.0%	0.83 [0.44, 1.58]
Neeki 2018	293	362	293	362	2.9%	1.00 [0.69, 1.45]
Ng 2019	54	67	42	50	0.5%	0.79 [0.30, 2.08]
Rivas 2021	480	887	361	446	11.3%	0.28 [0.21, 0.36]
Swendsen 2013	37	52	49	74	0.6%	1.26 [0.58, 2.72]
Valle 2014	128	150	129	150	1.0%	0.95 [0.50, 1.81]
Wafaisade 2016	187	258	187	258	2.6%	1.00 [0.68, 1.47]
**Total (95% CI)**		**13564**		**13234**	**100.0%**	**0.92 [0.86, 0.98]**
Total events	11017		10945			

Heterogeneity: $Chi^2 = 97.70$, df = 14 (P < 0.00001); $I^2 = 86\%$
Test for overall effect: Z = 2.53 (P = 0.01)

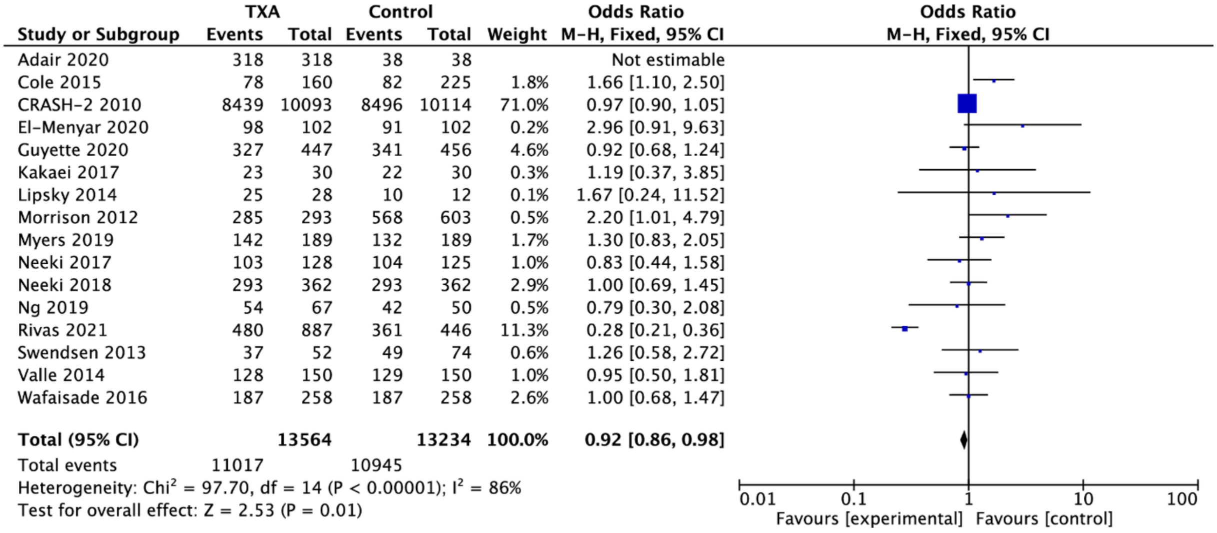

Figure S2. Forest plot of patient patients' sex (male) in TXA vs. Control group. The center of each square represents the weighted odds ratios for individual trials, and the corresponding horizontal line stands for a 95% confidence interval. The diamonds represent pooled results.

Study or Subgroup	TXA Mean	TXA SD	TXA Total	Non-TXA Mean	Non-TXA SD	Non-TXA Total	Weight	Mean Difference IV, Random, 95% CI
**3.4.1 Combat**								
Howard 2017	21.5	12.5	849	18.2	11.2	2924	9.0%	3.30 [2.37, 4.23]
Lipsky 2014	17.8	3.7	28	7	2.3	12	8.3%	10.80 [8.91, 12.69]
Morrison 2012	25.2	16.6	293	22.5	18.5	603	7.8%	2.70 [0.29, 5.11]
**Subtotal (95% CI)**			**1170**			**3539**	**25.1%**	**5.60 [0.68, 10.52]**
Heterogeneity: $Tau^2 = 18.04$; $Chi^2 = 51.17$, df = 2 ($P < 0.00001$); $I^2 = 96\%$								
Test for overall effect: Z = 2.23 (P = 0.03)								
**3.4.2 Civil**								
Cole 2015	33	13	160	29	10	225	7.8%	4.00 [1.60, 6.40]
El-Menyar 2020	24	0.8	102	24.99	0.9	102	9.2%	-0.99 [-1.22, -0.76]
Guyette 2020	13.25	2.8	447	12	3	456	9.2%	1.25 [0.87, 1.63]
Myers 2019	19.25	2.5	189	14.5	2.3	189	9.1%	4.75 [4.27, 5.23]
Neeki 2017	12.96	9.03	128	17	10.74	125	7.8%	-4.04 [-6.49, -1.59]
Neeki 2018	16.08	10.69	362	17.15	11.71	362	8.5%	-1.07 [-2.70, 0.56]
Ng 2019	27	16	67	25	16	50	4.5%	2.00 [-3.86, 7.86]
Swendsen 2013	27.1	15	52	20.5	16.8	74	4.7%	6.60 [1.01, 12.19]
Valle 2014	28	16	150	28	17	150	6.4%	0.00 [-3.74, 3.74]
Wafaisade 2016	24	14	258	24	16	258	7.6%	0.00 [-2.59, 2.59]
**Subtotal (95% CI)**			**1915**			**1991**	**74.9%**	**1.01 [-0.87, 2.88]**
Heterogeneity: $Tau^2 = 7.27$; $Chi^2 = 494.46$, df = 9 ($P < 0.00001$); $I^2 = 98\%$								
Test for overall effect: Z = 1.05 (P = 0.29)								
**Total (95% CI)**			**3085**			**5530**	**100.0%**	**2.17 [0.44, 3.90]**
Heterogeneity: $Tau^2 = 8.46$; $Chi^2 = 648.46$, df = 12 ($P < 0.00001$); $I^2 = 98\%$								
Test for overall effect: Z = 2.46 (P = 0.01)								
Test for subgroup differences: $Chi^2 = 2.92$, df = 1 (P = 0.09), $I^2 = 65.8\%$								

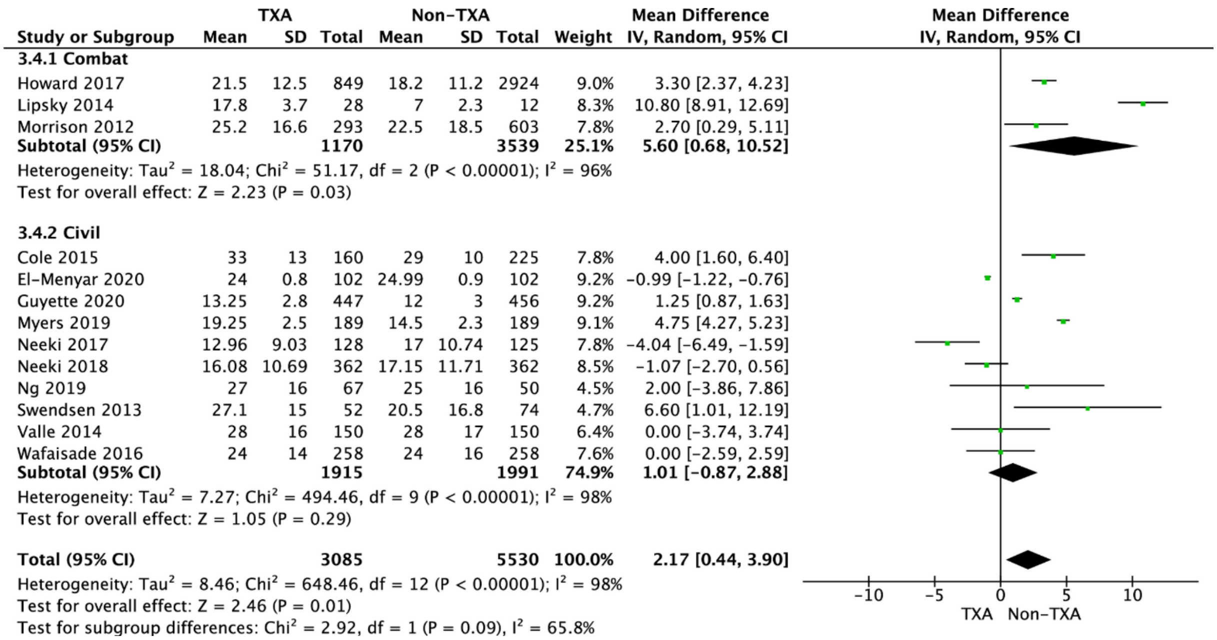

Figure S3. Forest plot of injury severity score at admission in TXA vs. Control group. The center of each square represents the weighted mean differences for individual trials, and the corresponding horizontal line stands for a 95% confidence interval. The diamonds represent pooled results.

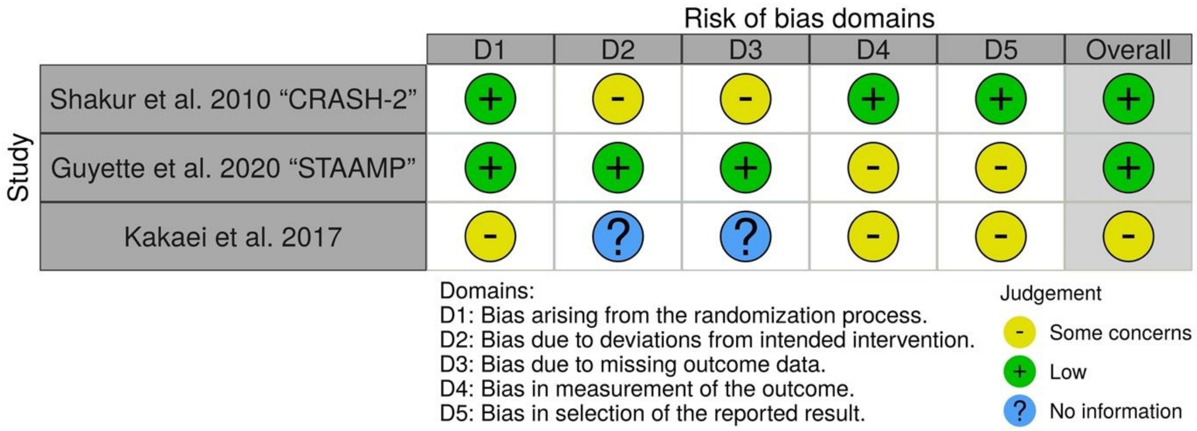

Figure S4. A summary table of review authors' judgements for each risk of bias item for each randomized study.

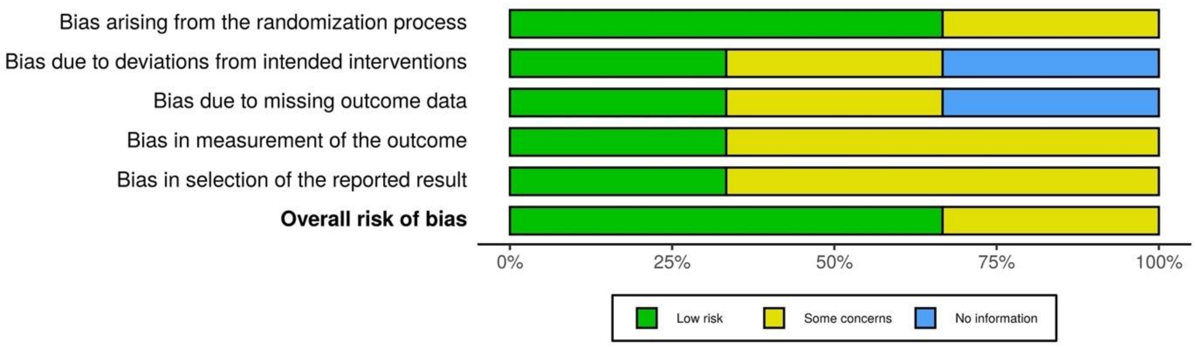

Figure S5. A plot of the distribution of review authors' judgements across randomized studies for each risk of bias item.

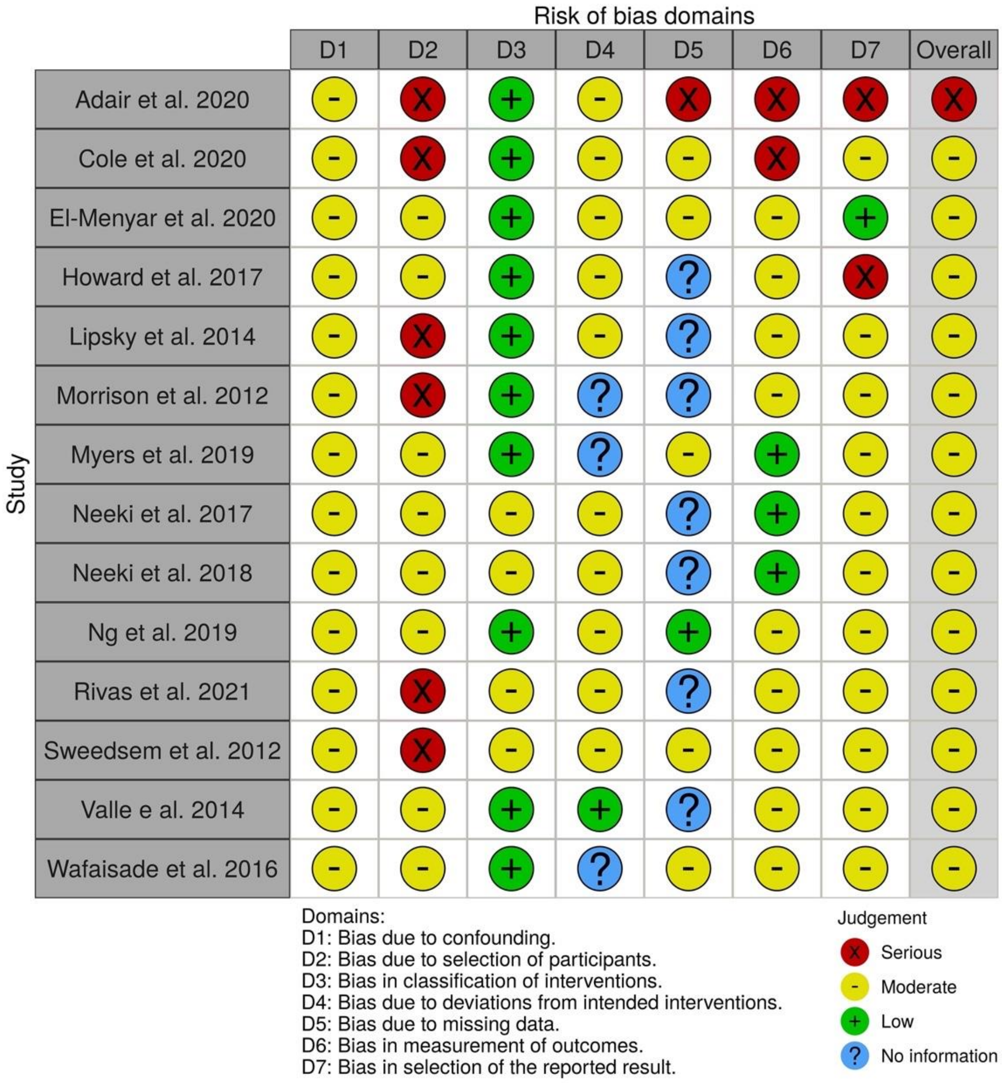

Figure S6. A summary table of review authors' judgements for each risk of bias item for each non-randomized study.

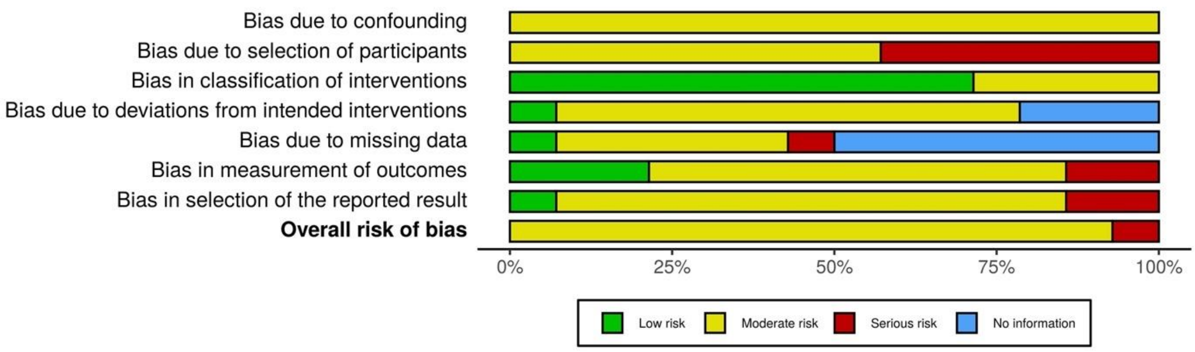

Figure S7. A plot of the distribution of review authors' judgements across non-randomized studies for each risk of bias item.
